# Evidence gaps among systematic reviews examining the relationship of race, ethnicity, and social determinants of health with adult inpatient quality measures

**DOI:** 10.1017/ash.2024.397

**Published:** 2024-09-23

**Authors:** Sonali D. Advani, Alison G. Smith, Ibukunoluwa C. Kalu, Reinaldo Perez, Stephanie Hendren, Raymund B. Dantes, Jonathan R. Edwards, Minn Soe, Sarah H. Yi, Janine Young, Deverick J. Anderson

**Affiliations:** 1 Duke Center for Antimicrobial Stewardship and Infection Prevention, Durham, NC, USA; 2 Division of Infectious Diseases, Department of Medicine, Duke University School of Medicine, Durham, NC, USA; 3 Department of Medicine, Duke University School of Medicine, Durham, NC, USA; 4 Division of Pediatric Infectious Diseases, Department of Pediatrics, Duke University School of Medicine, Durham, NC, USA; 5 Duke University Medical Center Library, Durham, NC, USA; 6 Division of Healthcare Quality Promotion, National Center for Emerging and Zoonotic Infectious Diseases, Centers for Disease Control and Prevention, Atlanta, GA, USA; 7 Division of Hospital Medicine, Department of Medicine, Emory University School of Medicine, Atlanta, GA, USA; 8 Division of Academic General Pediatrics, Department of Pediatrics, University of California San Diego School of Medicine, San Diego, CA, USA

## Abstract

**Background:**

The field of healthcare epidemiology is increasingly focused on identifying, characterizing, and addressing social determinants of health (SDOH) to address inequities in healthcare quality. To identify evidence gaps, we examined recent systematic reviews examining the association of race, ethnicity, and SDOH with inpatient quality measures.

**Methods:**

We searched Medline via OVID for English language systematic reviews from 2010 to 2022 addressing race, ethnicity, or SDOH domains and inpatient quality measures in adults using specific topic questions. We imported all citations to Covidence (www.covidence.org, Veritas Health Innovation) and removed duplicates. Two blinded reviewers assessed all articles for inclusion in 2 phases: title/abstract, then full-text review. Discrepancies were resolved by a third reviewer.

**Results:**

Of 472 systematic reviews identified, 39 were included. Of these, 23 examined all-cause mortality; 6 examined 30-day readmission rates; 4 examined length of stay, 4 examined falls, 2 examined surgical site infections (SSIs) and one review examined risk of venous thromboembolism. The most evaluated SDOH measures were sex (n = 9), income and/or employment status (n = 9), age (n = 6), race and ethnicity (n = 6), and education (n = 5). No systematic reviews assessed medication use errors or healthcare-associated infections. We found very limited assessment of other SDOH measures such as economic stability, neighborhood, and health system access.

**Conclusion:**

A limited number of systematic reviews have examined the association of race, ethnicity and SDOH measures with inpatient quality measures, and existing reviews highlight wide variability in reporting. Future systematic evaluations of SDOH measures are needed to better understand the relationships with inpatient quality measures.

## Background

Social determinants of health (SDOH) such as income, access to health care and education can exacerbate health inequities. Recent data have shown that over a third of Americans reported challenges with meeting their basic social needs, such as stable housing, adequate food, and reliable transportation.^1^ SDOH may affect various health outcomes that have important implications for healthcare cost and quality, including timing of disease diagnosis, patient use of healthcare services, timely access to sub-specialty referrals, diagnostic studies, surgical interventions, and hospital admissions/readmissions. Specifically, high-value healthcare metrics must incorporate these SDOH measures, and quality improvement interventions should demonstrate their impact on health inequities.^2^

In June 2021, the Board of Directors of the Association for Professionals in Infection Prevention and Epidemiology (APIC) commissioned a task force to evaluate the landscape of social and health inequities in hospital infection prevention using the principles of the Biopsychosocial Model and SDOH.^3^ The APIC task force recommended required reporting of key demographic data elements, including ethnicity and race, to Department of Health and Human Services.^4^ As populations, communities and contexts differ, SDOHs of importance may also differ. Despite the development of screening tools for SDOH, there is significant variation in these measures and in the capacity to integrate screening into current care procedures. EHR software providers and national SDOH experts recognize the absence of standardized definitions and methods for documenting and reporting SDH in EHRs as obstacles to implementation.^5^

We assessed recent systematic reviews examining the relationship between race, ethnicity, SDOH and inpatient quality measures to better understand the gaps in current evidence.

## Methods

### Review process

We searched Medline via OVID for English language systematic reviews from 2010 to 2022 addressing race, ethnicity, or SDOH domains (Table 1) and 29 inpatient quality measures (Table 2) using six specific topic questions (Table 3). Search terminology and logic used for narrative review are described in Supplement 1. In our narrative search, we used the Healthy People 2030 and Kaiser Family Foundation frameworks to define a list of SDOH topics or possible terms. We imported all citations to Covidence (www.covidence.org, Veritas Health Innovation) and removed duplicates. Two blinded reviewers assessed all systematic reviews for inclusion in 2 phases: (1) title/abstract review, followed by (2) full-text review. Articles were included if they discussed race, ethnicity or other author predefined SDOH search terms and inpatient quality measures (listed in Table 2). Discrepancies were resolved by a third reviewer. Due to limited data available for Question IV (How often are patients admitted to the hospital intentionally screened for SDOH measures?), the authors performed an additional targeted search of the literature for research on screening for SDOH. Pediatric studies were excluded due to the focus on adult inpatient quality measures. Terms used in this report reflect the terminology from included reviews (eg, “non-white race”).

**Table 1.** Social determinant of health measures included in narrative review search*

Economic stability	Neighborhood environment	Education	Food	Community and social context	Healthcare system
Income	Geography or Zip Code	Childhood education	Hunger	Social integration	Health coverage
Medical Bills	Environment or Pollution	Higher education	Access to healthy options	Support Systems	Clinician availability
Socio-economic Status	Safety	Literacy	Obesity	Community engagement	Clinician linguistic and cultural competency
Debt	Parks	Vocational training		Age Sex or gender	Veterans or VA
Employment	Walkability			Stress	Quality of Care
Support	Playgrounds			Discrimination	Insured/insurance
Expenses	Transportation			Sexuality Sexual orientation	
				Abuse/neglect	

* We used Healthy People 2030 and Kaiser Family Foundation frameworks to define a list of SDOH terms^38,39^

**Table 2.** Four domains of inpatient quality measures that were included in the narrative review search^40^

Domain	Inpatient quality measures
General Quality Measures	30-day mortality
	All-cause mortality
	30-day readmission
	Hospital length of stay
	Antibiotic utilization
	Admit decision time to emergency department departure time
Unintended Complications of Healthcare	Hypoglycemia
	Severe sepsis and septic shock bundle
	In-hospital fall rate
	Pressure ulcer rate
	Iatrogenic pneumothorax rates
	Deep vein thrombosis
	Other catheter complications
Medication Use	High risk medication use in elderly
	Eligible patients discharged on statin medication
	Eligible patients discharged on antithrombotic therapy
	Anticoagulation therapy for atrial fibrillation/flutter
	Antithrombotic therapy by the end of Hospital Day 2
	Incomplete medication documentation
	Venous Thromboembolism (VTE) prophylaxis
	Intensive care unit VTE prophylaxis
	New opioid prescription
	Safe use of opioids
Healthcare-Associated Infections	Central line associated bloodstream infections
	Catheter associated urinary tract infections
	Surgical site infections
	Hospital-onset *C. difficile* infections
	Methicillin-resistant *Staphylococcus aureus* bacteremia
	Hospital-onset bloodstream infections

**Table 3.** Topic questions used to guide the narrative review

Topic areas	Topic questions
**I. Association with Quality Measures**	How are race, ethnicity and Social Determinants of Health SDOH associated with inpatient quality measures?
**II. Definitions**	How are race, ethnicity, and SDOH measures defined in the context of inpatient quality measures?
**III. Documentation**	How complete and accurate are documentation of race and ethnicity in observational health databases, including electronic health records (EHRs)? What SDOH data elements are included in EHRs and observational health databases?
**IV. Admission Screening**	How often are patients admitted to the hospital screened for SDOH measures?
**V. Risk Stratification**	How often are patients identified as having one or more social risk factor?
**VI. Range of Uses**	How are race, ethnicity and SDOH measures reported in context of inpatient quality measures?

### Assessment of quality

We used the Scale for the Assessment of Narrative Review Articles (SANRA) scale for assessment of the quality of narrative review (Supplement 2).^6^

## Results and discussion

Of 480 systematic reviews identified, 8 duplicates were removed, and 125 abstracts were selected for full-text review. These 125 systematic reviews were assessed, and 39 reviews (9%) were included in the final analysis (Figure 1). Our inclusion percentage is in line with high quality systematic reviews (2–8%).^7^ According to the SANRA scale, the quality of this narrative review was very high (score:10, Supplement 2). Below are summarized responses to the questions that guided our search strategy:

1. **I.** **What is the association of race, ethnicity, SDOH with quality measures? (n = 23)** A total of 39 articles included data regarding the association of race, ethnicity, and SDOH with six specific inpatient quality measures (Table 4). We did not identify articles that addressed the remaining 23 inpatient quality measures (Table 2).
  - **30-day readmission:** Six reviews evaluated the association of race, ethnicity, or SDOH with 30-day readmission rates. Age (n = 4), sex (n = 4), race (n = 3), and income (n = 3) were most evaluated. Of these reviews, the majority demonstrated that patients with increasing age, male sex, and “non-white race” (as reported in systematic review) had increased risk of 30-day readmissions. One review evaluating inpatients admitted with cardiac conditions concluded that female sex was associated with increased risk of 30-day readmission and that female inpatients were less likely to receive a cardiac intervention compared to male patients.^8^ Some reviews concluded that public insurance (n = 2), obesity (n = 1), and unemployment status (n = 1) were associated with increased risk of 30-day readmission. Additionally, one review concluded that higher income was associated with a decreased risk of *any* hospital readmission (ie, not limited to all-cause 30-day readmission) while lower levels of social integration, including participation in a broad range of social relationships as measured by the Social Network Index, was associated with an increased risk of *any* type of hospital readmission.^9^
  - **All-cause mortality:** Twenty-three reviews evaluated the association of race, ethnicity, or SDOH with all-cause mortality. The most evaluated measures were sex (n = 6), race (n = 5), exercise (n = 5), and nutrition (n = 3).^10^ Among these reviews, the majority demonstrated male sex, “non-white race,” and frailty were associated with increased risk of all-cause mortality, while increased physical activity and improved nutrition were associated with decreased risk. A smaller number of reviews concluded that poverty, higher levels of pollution exposure (particulate matter), lower socioeconomic status, or immigrant status were associated with increased risk. A single review concluded that age >75 years was associated with increased risk,^11^ while a different individual review concluded that higher level of employment was associated with a decreased risk.^12^
  - **Falls:** Four reviews evaluated the association of race, ethnicity, or SDOH with inpatient falls. All four evaluated the association of exercise with falls; the majority concluded that increased exercise and physical activity were associated with decreased risk of falls, including both inpatient falls and falls in all settings. A single review concluded that use of alcohol or other drug use was associated with increased risk of falls.^13^ Similarly, a different review concluded that language barriers and/or a communication disability were associated with increased risk of falls among neurology patients.^14^ One review was inconclusive about the association of nutrition and vitamin D levels with the risk of falls.^15^
  - **Length of stay:** Three reviews evaluated the association of race, ethnicity, or SDOH with hospital length of stay. Two reviews concluded that “non-white race” was associated with a longer length of stay.^16,17^ Similarly, a single review concluded that lower levels of social integration were associated with longer length of stay.^18^
  - **Surgical Site Infections (SSI):** Two reviews evaluated the association of race, ethnicity, or SDOH with SSIs. One review concluded that “non-white race” was associated with increased risk of SSI.^17^ A separate review concluded that obesity was associated with SSI.^19^
  - **Venous thromboembolism (VTE):** One review evaluated the association of race, ethnicity, or SDOH with risk of VTE.^20^ This single review concluded that increasing age, increasing BMI, female sex, and African American race were associated with increased risk of VTE.
  **Figure 1.**
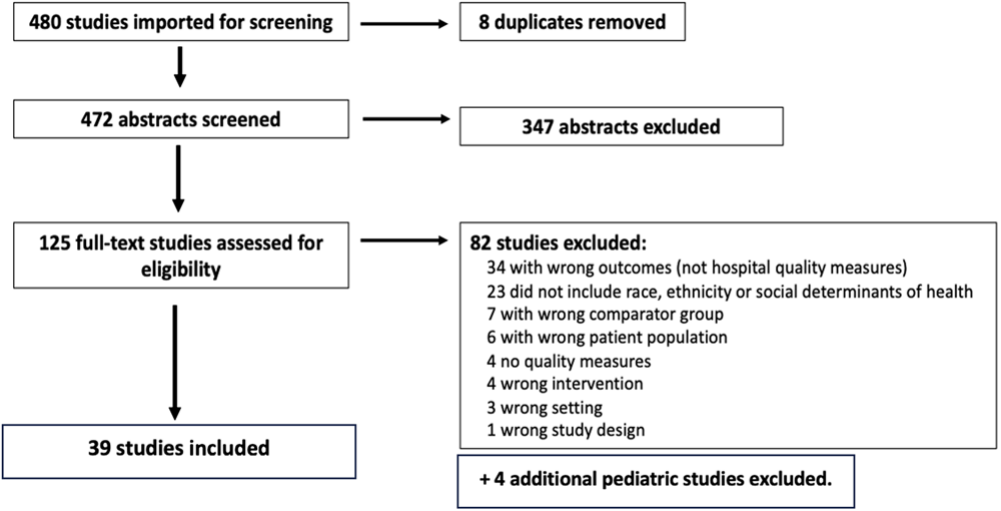
Flow diagram of the narrative review process.
  **Table 4.** Narrative review of existing systematic reviews describing associations between race, ethnicity, social determinants of health and quality measures, 2010–2022, N = 39

	30-day readmission	All-cause mortality	Falls	Length of hospital stay	Surgical site infections	Venous thromboembolism
Race/Ethnicity*	**↑** risk if non-white race* (n = 3)	**↑** risk if non-white race* (n = 5)		Non-white race* associated with longer stay (n = 1)	↑ risk In African American compared to white (n = 1)	African American race with increased risk compared to white (n = 1)
Sex and Age	↑ with age (n = 4) ↑ for males (n = 4) ↑ for women with cardiac symptoms (n = 1)	↑ risk if >75 years of age (n = 1); Mixed results of effect of sex (n = 6)				
Economic Stability	↑ with unemployment (n = 1); No clear evidence of income level on 30-day readmission (n = 3)	↓ with employment (n = 1); ↓ with higher income (n = 1) ↑ with low socioeconomic status* or poverty (n = 2)				
Neighborhood & Physical Environment	No impact of number inhabitants in the household (n = 1)	↑ with air pollution (n = 1)				
Education	No overall impact of education level (n = 2)	Mixed evidence (n = 3)	↑ with communication disability (n = 1)			
Food and Nutrition	↑ if obesity at index admission (n = 1); ↑ if dehydration at index admission (n = 1)	Mixed results of effect of obesity (n = 2); Improved nutrition decreases risk (n = 1)	Mixed results of effect of Vitamin D level (n = 1)	↑ with malnutrition (n = 1)	↑ risk with higher body mass index (BMI, n = 1)	↑ risk with higher BMI (n = 1)
Community Social context	No overall impact of living alone (n = 1)	↑ with history of abuse (n = 1); ↑ with refugee status (n = 1)	↑ with alcohol or other drug use (n = 1)	↑ with lower community social integration (n = 1)		
Health System	↑ with public insurance (n = 2)	Mixed evidence on effect of private insurance (n = 2)				

* Reported in table as defined/reported in systematic review
2. **II.** **How are race, ethnicity, and SDOH measures defined in the context of healthcare quality? (n = 10)**

Among the 39 systematic reviews, 10 reviews included definitions for race, ethnicity or SDOH and examined their association with inpatient quality measures, specifically all-cause mortality and alcohol-attributable mortality. In the reviews that clearly defined SDOH measures, there was a heavy reliance on available metrics of income, insurance, poverty indices, education, housing, and crime to define socioeconomic statuses.^21–27^ Specifically, reviews focused solely on single measures when it related to frailty or migration. However, reviews that used socioeconomic status measures typically included multiple measures (eg, income, residence, and insurance type, etc.). To facilitate comparisons, these reviews restricted their included studies to either a single SDOH measure or to their predetermined SDOH definition, in particular for socioeconomic status.

Race and ethnicity categorizations were either not clearly defined or variably defined often relying on U.S. Census Bureau definitions or other similar agencies.^27^ For example, in an included review of “indigenous” adults with rheumatic diseases, “indigenous” was defined as Canadian First Nations, Native Americans and Australian Aborigine to account for populations across Canada, Australia, and the United States.^26^ In another study on infant mortality among immigrants, country of birth was used as a proxy for race while ethnicity was reported as an “undefined term” representing national origin or citizenship.^25^ Also of note, in a number of reviews, SDOH factors with subjective definitions were used. For example, Valtorta et al. evaluated the social relationships of older adults by defining categories of isolation and loneliness based upon availability of perceived support.^18^

1. **III.** **How complete and accurate is documentation of race and ethnicity and SDOH in observational health databases, including EHRs? (n = 3)**

While basic demographic data (eg, age, race, ethnicity, insurance type) are often included as part of routine EHRs to facilitate medical billing and/or to fulfill federal reporting requirements, only three reviews assessed data completeness, while no reviews assessed data quality. One study evaluating patients older than 15 years of age presenting to the hospital with falls noted a range of 1–88% of patient records had missing documentation for alcohol use or alcohol consumption.^13^ Despite the lack of “best practices” to address data completeness and accuracy, one review by Cook, et al. sought to assess patient-level data in EHRs.^28^ Their review searched for race, ethnicity, language preference, health insurance status, country of origin, occupation, socioeconomic status, education level, environmental health determinants (eg, proximity to food sources, walkability, exposure to toxins), and geocoded patient address data as it was documented in the EHR. Their analyses revealed higher rates of race and ethnicity misclassification and poor data quality for some racial groups. Specifically, assumptions were made by data recorders around the ethnicity of patients with “Spanish sounding” last names who were, in fact, neither Hispanic nor Latino, as well as low data accuracy for patients identified as having “Hispanic or Latino” ethnicity in general.^28^

Among the included systematic reviews, few included any documented measures of disability, information on socioeconomic status, language, interpreter use, literacy, or numeracy data. Overall, there appears to be minimal consensus on which SDOH measures to collect besides those required for federal reporting and/or billing purposes. To account for variation in available EHR documentation, many reviews included external data sources such as census data or aggregate zip code information which are approaches with several known limitations. These external data sources lose key patient-level data in the aggregation process, may solely focus on income or education as proxy measures of SDOH, and may not adequately capture intersectionality among SDOH measures.^29^

1. **IV.** **How often are patients admitted to the hospital intentionally screened for SDOH measures? (n = 0)** No systematic reviews addressed this question. An additional targeted search of the literature for research on screening for SDOH revealed the following information:
2. **Screening tools:** A wide variety of published tools exist for potential use in hospital screening, each with distinct lengths, modes of administration and areas of focus.^30^ For example, the Protocol for Responding to and Assessing Patient’s Risks, Assets, and Experiences is a tool developed for and by community health centers, is available in over 30 languages (pending validation in these languages), and is increasingly used across healthcare settings.^31^ The Accountable Health Communities Health-Related Social Needs screening tool was developed with expert input and tested by the Centers for Medicare & Medicaid Services for use in a range of clinical delivery settings.^32^ The Institute of Medicine has identified SDOH domains for the meaningful use of EHRs, and the Office of the National Coordinator for Health Information Technology (ONC) has established interoperability standards to facilitate the sharing of SDOH data elements.^33^ With this in place, there is potential for wider implementation of SDOH screening programs facilitated through EHRs. However, there are no agreed-upon national standards for which data elements should be routinely collected or how these data should be applied to achieve meaningful use. A recent scoping review revealed the most frequently assessed SDOHs in studies limited to inpatient general internal medicine wards are food security, finances, housing and transportation.^30^ Similarly, there was a lack of current standard on the best methods of administering these screening tools, based on preferred language spoken, literacy, and/or education level; existing reviews utilized data collected by self-report with a written questionnaire (which excludes other language speakers, vision-impaired, and preliterate patients), physician administered, or administration by dedicated staff (with no documentation of interpreter use for other language speakers). Previous reviews have demonstrated that patient responses are influenced by fears around the implications of this data collection.^34^
3. **Implementation of screening and interventions:** Most of the available literature consists of implementation reviews assessing the introduction of a SDOH screening tool to a specific clinical site. The majority of these reviews were performed in the outpatient setting and showed that implementation of these screenings was feasible with large numbers of unmet patient needs identified.^35^ Ability to address identified needs, however, varied greatly by the resources available in the given clinical context. There are limited number of systematic reviews looking at universal implementation of adult inpatient screening for SDOH. One systematic review identified only 8 studies, of which 5 interviewed patients directly, all in English only. The largest of these reviews included approximately 1400 patients, a small sample size.^30^ Healthcare system resources needed to implement systematic screening and SDOH barrier interventions were not addressed in these reviews.
4. **V.** **How often are patients identified as having one or more SDOH risk factor?(n = 0)**

Our search strategy failed to identify any published systematic reviews to address the prevalence of SDOH risk factors in hospitalized patients and sub-populations. Given that universal inpatient SDOH screening has not been implemented, accurate estimates of risk factor identification are not available. Based on articles identified to address screening implementation (see Question IV, above), estimates were available from small reviews. In these small reviews, the rates of identified SDOH risk factors were highly variable depending on the screening tool used and the underlying population assessed. Two of the largest reviews are summarized, below, demonstrating this variability.

Meyer et al. looked at implementation of universal screening for SDOH at New York-Presbyterian Hospital and associated primary care sites.^36^ Over the review period, 13,273 patients in North Manhattan were screened; 82% identified as Hispanic, 14% identified as Black/African American and the average household income was $24,000. In this population, 27% screened positive for food insecurity, 25% screened positive for housing insecurity, 12% screened positive for transportation needs, 8% screened positive for utility needs and 1% screened positive for safety needs. Smith et al. analyzed 1,427 patients admitted to the general medicine service at an academic health center in Toronto, Canada.^37^ Their review population was less racially diverse (69% white, 11% Asian, 5% Black, 14% “other”). Income data were limited (nearly 50% of the population selected “prefer not to answer”; 14% reported an income below $20,000 USD; 12% reported challenges with English language that inhibited their care; 54% reported having at least one disability. Food and housing security, transportation needs, and safety needs were not addressed in this review.

1. **VI.** **How often are race, ethnicity and SDOH measures reported when describing specific hospital quality measures?**

The most evaluated demographic and SDOH measures were sex (n = 9), income and/or employment status (n = 9), age (n = 6), race (n = 6), and education (n = 5). We found very limited assessment of other SDOH measures such as economic stability, neighborhood, language spoken, and health systems access in the included systematic reviews. SDOH measures were most assessed related to the hospital quality measures of all-cause mortality and readmission rates; few addressed increased risk for falls, venous thromboembolism, or length of stay; no systematic reviews assessed medication use errors or healthcare-associated infections (HAIs) besides SSIs (Table 4).

## Limitations

This review only included systematic reviews published since 2010 and excluded pediatric literature. As our review was limited to systematic reviews, our conclusions will not reflect all conducted research. In our search strategy centered around identifying articles that identified both SDOH and inpatient quality measures. As a result, the identified literature described the circumstances of very specific populations (eg, patients admitted for acute myocardial infarction in adults), and did not necessarily reflect the larger hospital population. Many of the included reviews relied on retrospective data abstraction from the EHR, instead of self-reported or directly reported data from patients. Additionally, many of these systematic reviews assessing race, ethnicity, and SDOH in the context of inpatient quality measures is quite recent, leaving a gap in knowledge about previous practices. our narrative search, the use of Healthy People 2030 and Kaiser Family Foundation frameworks to define a list of SDOH topics or possible terms was not exhaustive; therefore, we may have excluded reviews that addressed other SDOH indicators not included in these frameworks.

## Conclusions

A limited number of systematic reviews have examined the association of race, ethnicity and SDOH measures with inpatient quality measures. Among recent systematic reviews, there was wide variability and lack of standardization of which SDOH indicators to include as well as how race, ethnicity, and specific SDOH are collected and reported in EHRs. Given the highly variable nature of local epidemiology and case mix, screening tools used, and screening implementations, it is difficult to draw conclusions about our ability to accurately identify patients with SDOH risk factors. These gaps substantially limit interpretation of available patient-level data. Existing systematic reviews also had very limited information on the state of hospital-based screening for patients for SDOH measures and social risk factors. To date, SSI is the only HAI for which there are published systematic reviews evaluating its association with race, ethnicity, and SDOH.

Efforts are needed to improve existing SDOH screening questionnaires, validate these questions in multiple languages, and determine the best approaches for administration (eg, written, verbal, prerecorded, etc.). Once these validated SDOH questionnaires are identified and scripted, hospital systems will need to pilot standardized approaches for implementation. Our next steps include performing a systematic review to examine the association of race, ethnicity and SDOH measures with HAIs. Knowledge gained from these reviews will help to identify strategies for reporting SDOH terms in relation to specific HAI measures.

## Supporting information

Advani et al. supplementary material 1 Advani et al. supplementary material

Advani et al. supplementary material 2 Advani et al. supplementary material

## Supplementary material

To view supplementary material for this article, please visit https://doi.org/10.1017/ash.2024.397

## References

[1] Gordon NP , Shanks CB , Grant RW. Social risks, social needs, and attitudes toward social health screening 1 year into the COVID-19 pandemic: survey of adults in an integrated health care delivery system. Perm J 2023;27:61–74. 10.7812/TPP/22.142 37063058 PMC10266851

[2] Dzau VJ , Mate K , O’Kane M. Equity and quality-improving health care delivery requires both. JAMA 2022;327:519–520. 10.1001/jama.2022.0283 35060998

[3] Nix CD , Bubb TN , Maddox VB. Recommendations from the association for professionals in infection control and epidemiology health inequalities & disparities task force. Am J Infect Control 2023;51:107–109. 10.1016/j.ajic.2022.10.004 36257494

[4] McGrath C , Deloney V , Logan L , et al. Monitoring disparities in healthcare-associated infection surveillance: a SHEA Research Network (SRN) Survey. Antimicrob Steward Healthc Epidemiol 2022;2(S1):s82–s83. 10.1017/ice.2023.181 PMC11007321 37700531

[5] Freij M , Dullabh P , Lewis S , Smith SR , Hovey L , Dhopeshwarkar R. Incorporating social determinants of health in electronic health records: qualitative study of current practices among top vendors. JMIR Med Inform 2019;7:e13849. 10.2196/13849 31199345 PMC6592390

[6] Baethge C , Goldbeck-Wood S , Mertens S. SANRA-a scale for the quality assessment of narrative review articles. Res Integr Peer Rev 2019;4:5. 10.1186/s41073-019-0064-8 30962953 PMC6434870

[7] Wang Z , Nayfeh T , Tetzlaff J , O’Blenis P , Murad MH. Error rates of human reviewers during abstract screening in systematic reviews. PLoS One 2020;15:e0227742. 10.1371/journal.pone.0227742 31935267 PMC6959565

[8] Hoang-Kim A , Parpia C , Freitas C , et al. Readmission rates following heart failure: a scoping review of sex and gender based considerations. BMC Cardiovasc Disord 2020;20:223. 10.1186/s12872-020-01422-3 32408892 PMC7222562

[9] Desai MM , Stauffer BD , Feringa HHH , Schreiner GC. Statistical models and patient predictors of readmission for acute myocardial infarction: a systematic review. Circ Cardiovasc Qual Outcomes 2009;2:500–507. 10.1161/CIRCOUTCOMES.108.832949 20031883

[10] Cohen S , Doyle WJ , Skoner DP , Rabin BS , Gwaltney JM, Jr. Social ties and susceptibility to the common cold. JAMA 1997;277:1940–1944. 9200634

[11] Damayanthi HDWT , Prabani KIP , Weerasekara I. Factors associated for mortality of older people with COVID 19: a systematic review and meta-analysis. Gerontol Geriatr Med 2021;7:23337214211057392. 10.1177/23337214211057392 34888405 PMC8649451

[12] Tao S , Zeng X , Liu J , Fu P. Socioeconomic status and mortality among dialysis patients: a systematic review and meta-analysis. Int Urol Nephrol 2019;51:509–518. 10.1007/s11255-019-02078-5 30689180

[13] Lau G , Ang JY , Kim N , et al. Prevalence of alcohol and other drug use in patients presenting to hospital for fall-related injuries: a systematic review. Inj Prev 2022;28:381–393. 10.1136/injuryprev-2021-044513 35508365

[14] Hemsley B , Steel J , Worrall L , et al. A systematic review of falls in hospital for patients with communication disability: highlighting an invisible population. J Saf Res 2019;68:89–105. 10.1016/j.jsr.2018.11.004 30876524

[15] Guirguis-Blake JM , Michael YL , Perdue LA , Coppola EL , Beil TL. Interventions to prevent falls in older adults: updated evidence report and systematic review for the US preventive services task force. JAMA 2018;319:1705–1716. 10.1001/jama.2017.21962 29710140

[16] Mendoza J , Pangal DJ , Cardinal T , et al. Systematic review of racial, socioeconomic, and insurance status disparities in neurosurgical care for intracranial tumors. World Neurosurg 2022;158:38–64. 10.1016/j.wneu.2021.10.126 34710578

[17] Mo K , Ikwuezunma I , Mun F , et al. Racial disparities in spine surgery: a systematic review. Clin Spine Surg 2023; 36(6):243–252. 10.1097/BSD.0000000000001383 35994052

[18] Valtorta NK , Moore DC , Barron L , Stow D , Hanratty B. Older adults’ social relationships and health care utilization: a systematic review. Am J Public Health 2018;108:e1–e10. 10.2105/AJPH.2017.304256 PMC5844393 29470115

[19] Partha Sarathi CI , Mowforth OD , Sinha A , et al. The role of nutrition in degenerative cervical myelopathy: a systematic review. Nutr Metabol Insights 2021;14:11786388211054664. 10.1177/11786388211054664 PMC8558601 34733105

[20] Barrett MC , Whitehouse MR , Blom AW , Kunutsor SK. Host-related factors for venous thromboembolism following total joint replacement: a meta-analysis of 89 observational studies involving over 14 million hip and knee replacements. J Orthop Sci 2020;25:267–275. 10.1016/j.jos.2019.04.003 31029528

[21] Probst C , Roerecke M , Behrendt S , Rehm J. Gender differences in socioeconomic inequality of alcohol-attributable mortality: a systematic review and meta-analysis. Drug Alcohol Rev 2015;34:267–277. 10.1111/dar.12184 25109218

[22] Probst C , Kilian C , Sanchez S , Lange S , Rehm J. The role of alcohol use and drinking patterns in socioeconomic inequalities in mortality: a systematic review. Lancet Pub Health 2020;5:e324–e332. 10.1016/S2468-2667(20)30052-9 32504585

[23] Ornaghi PI , Afferi L , Antonelli A , et al. Frailty impact on postoperative complications and early mortality rates in patients undergoing radical cystectomy for bladder cancer: a systematic review. Arab J Urol 2020;19:9–23. 10.1080/2090598X.2020.1841538 33763244 PMC7954492

[24] Mustard CA , Etches J. Gender differences in socioeconomic inequality in mortality. J Epidemiol Comm Health 2003;57:974–980. 10.1136/jech.57.12.974 PMC1732354 14652265

[25] Gissler M , Alexander S , MacFarlane A , et al. Stillbirths and infant deaths among migrants in industrialized countries. Acta Obstet Gynecol Scand 2009;88:134–148. 10.1080/00016340802603805 19096947

[26] Hurd K , Barnabe C. Mortality causes and outcomes in Indigenous populations of Canada, the United States, and Australia with rheumatic disease: a systematic review. Semin Arthritis Rheum 2018;47:586–592. 10.1016/j.semarthrit.2017.07.009 28823732

[27] Cusimano MD , Pshonyak I , Lee MY , Ilie G. A systematic review of 30-day readmission after cranial neurosurgery. J Neurosurg 2017;127:342–352. 10.3171/2016.7.JNS152226 27767396

[28] Cook LA , Sachs J , Weiskopf NG. The quality of social determinants data in the electronic health record: a systematic review. J Am Med Inform Assoc 2021;29:187–196. 10.1093/jamia/ocab199 34664641 PMC8714289

[29] Mahmoudi E , Kamdar N , Kim N , Gonzales G , Singh K , Waljee AK. Use of electronic medical records in development and validation of risk prediction models of hospital readmission: systematic review. BMJ 2020;369:m958. 10.1136/bmj.m958 32269037 PMC7249246

[30] Davis VH , Rodger L , Pinto AD. Collection and use of social determinants of health data in inpatient general internal medicine wards: a scoping review. J Gen Intern Med 2023;38:480–489. 10.1007/s11606-022-07937-z 36471193 PMC9905340

[31] National Association of Community Health Centers INAoAPCHOA. PRAPARE Protocol for Responding to and Assessing Patients’ Assets, Risks, and Experiences. Accessed July 1, 2023. https://prapare.org/

[32] Billioux A , Verlander K , Anthony S , Alley D. Standardized screening for health-related social needs in clinical settings: the accountable health communities screening tool. Discussion Paper. NAM Perspect 2017. https://nam.edu/standardized-screening-for-health-related-social-needs-in-clinical-settings-the-accountable-health-communities-screening-tool/

[33] HealthIT.gov. Social Determinants of Health. Accessed February 5, 2024. https://www.healthit.gov/health-equity/social-determinants-health

[34] Pinto AD , Glattstein-Young G , Mohamed A , Bloch G , Leung FH , Glazier RH. Building a foundation to reduce health inequities: routine collection of sociodemographic data in primary care. J Am Board Fam Med 2016;29:348–355. 10.3122/jabfm.2016.03.150280 27170792

[35] Bechtel N , Jones A , Kue J , Ford JL. Evaluation of the core 5 social determinants of health screening tool. Public Health Nurs 2022;39:438–445. 10.1111/phn.12983 34628675

[36] Meyer D , Lerner E , Phillips A , Zumwalt K. Universal screening of social determinants of health at a large US academic medical center, 2018. Am J Public Health 2020;110(S2):S219–S221. 10.2105/ajph.2020.305747 32663083 PMC7362698

[37] Smith RW , Kuluski K , Costa AP , et al. Investigating the effect of sociodemographic factors on 30-day hospital readmission among medical patients in Toronto, Canada: a prospective cohort study. BMJ Open 2017;7:e017956. 10.1136/bmjopen-2017-017956 PMC5728294 29237654

[38] DHHS. Healthy People 2030. Accessed October 14, 2022. https://health.gov/healthypeople

[39] KFF. Beyond Health Care: The Role of Social Determinants in Promoting Health and Health Equity. Accessed October 10, 2022. https://www.kff.org/racial-equity-and-health-policy/issue-brief/beyond-health-care-the-role-of-social-determinants-in-promoting-health-and-health-equity/

[40] CMS.gov. Quality Measures. Accessed Oct 10, 2023. https://www.cms.gov/medicare/quality/measures

