# Supplementary material for: Evidence gaps among systematic reviews examining the relationship of race, ethnicity, and social determinants of health with adult inpatient quality measures
Source: Antimicrob Steward Healthc Epidemiol. 2024 Sep 23;4(1):e139. doi: 10.1017/ash.2024.397 (PMC11427999; doi:10.1017/ash.2024.397)
Supplement: Advani et al. supplementary material 1 — Advani et al. supplementary material [file S2732494X24003978sup001.docx]

**Supplement 1: Search strategy**

**date filter: 2010 – November 2022

Research Questions Included:

How are race, ethnicity and SDOH measures reported when describing the impact on quality measures?

| **Set #** | **Search Strategy** | **Results** |
| --- | --- | --- |
| 1 | ("social determinant" OR "social determinants" OR race OR racial OR ethnicity OR ethnicities OR "minority group" OR "minority groups" OR sociodemographic OR socioeconomic OR "social class" OR "social factor" OR "social factors" OR "education level" OR "education levels" OR urban OR rural OR pollution OR immigration OR "undocumented immigrant" OR "undocumented immigrants" OR "drug use" OR "drug abuse" OR "abusing drugs" OR "alcohol use" OR alcoholic OR alcoholics OR incarceration OR incarcerated OR prisoner OR prisoners OR gender OR genders OR sexuality OR sexualities OR "sexual orientation" OR "sexual orientations" OR "military personnel" OR veteran OR veterans OR "economic stability" OR income OR housing OR workplace OR "work place" Or transportation OR "area index deprivation" OR geographic OR environment OR ability OR abilities OR walkability OR walkable OR park OR parks OR education OR language OR languages OR diet OR diets OR food OR foods OR family OR families OR community OR communities OR "social context" OR "social integration" OR women OR "mental health" OR insurance OR insured OR "provider availability" OR "social vulnerability index" OR "deprivation index").ti,ab. | 6434671 |
| 2 | (measure OR measures OR measurements OR measured).ti,ab. | 3510659 |
| 3 | (reporting OR reported).ti,ab. | 2421500 |
| 4  Quality outcomes of interest | (("central line" OR "central venous" OR "urinary catheter" OR "urinary catheters" OR "c diff" OR "clostridium difficile" OR "clostridioides difficile" OR healthcare OR "health care") adj2 (infection OR infections)).ti,ab. OR ("hospital onset bloodstream infection" OR "hospital-onset bloodstream infection" OR "hospital onset bloodstream infections" OR "hospital-onset bloodstream infections" OR "mrsa bacteremia" OR ("Staphylococcus aureus" AND resistant)).ti,ab. OR (CLABSI OR CLABSIs OR HAI OR HAIs OR CAUTI OR CAUTIs OR HOBSI OR HOBSIs OR HO-BSI OR HO-BSIs).ti. OR (mortality OR mortalities OR deaths OR hypoglycemia OR sepsis OR fall OR falls OR "pressure ulcer" OR "pressure ulcers" OR "iatrogenic pneumothorax" OR "deep vein thrombosis" OR "catheter complication" OR "catheter complications" OR "high risk medication use").ti. OR ((discharge OR discharging OR discharged) adj1 (statin OR statins OR antithrombotic)).ti,ab. OR ("anticoagulation therapy" OR "antithrombotic therapy").ti,ab. OR ((medication OR opioid OR antibiotic) adj (prescription OR prescriptions OR prescribed OR utilization)).ti,ab. OR ("venous thromboembolism prophylaxis").ti,ab. OR (length adj1 stay).ti,ab. OR (readmission OR readmissions).ti,ab. | 637549 |
| 5 | 1 AND 2 AND 3 AND 4 | 3823 |
| 6 | 5 AND ("systematic review".pt. OR ("systematic review" OR "systematic reviews" OR "scoping review" OR "scoping reviews" OR "umbrella review" OR "umbrella reviews").ti.) | 363 |

Research Questions Included:

How are race, ethnicity, and SDOH measures defined in the context of healthcare quality?

How often are patients admitted to the hospital screened for SDOH measures?

How often are patients who are screened for SDOH measures identified as having one or more social risk factor?

| **Set #** | **Search Strategy** | **Results** |
| --- | --- | --- |
| 1 | ("social determinant" OR "social determinants" OR race OR racial OR ethnicity OR ethnicities OR "minority group" OR "minority groups" OR sociodemographic OR socioeconomic OR "social class" OR "social factor" OR "social factors" OR "education level" OR "education levels" OR urban OR rural OR pollution OR immigration OR "undocumented immigrant" OR "undocumented immigrants" OR "drug use" OR "drug abuse" OR "abusing drugs" OR "alcohol use" OR alcoholic OR alcoholics OR incarceration OR incarcerated OR prisoner OR prisoners OR gender OR genders OR sexuality OR sexualities OR "sexual orientation" OR "sexual orientations" OR "military personnel" OR veteran OR veterans OR "economic stability" OR income OR housing OR workplace OR "work place" Or transportation OR "area index deprivation" OR geographic OR environment OR ability OR abilities OR walkability OR walkable OR park OR parks OR education OR language OR languages OR diet OR diets OR food OR foods OR family OR families OR community OR communities OR "social context" OR "social integration" OR women OR "mental health" OR insurance OR insured OR "provider availability" OR "social vulnerability index" OR "deprivation index").ti,ab. | 6646725 |
| 2  hospital admission | ("hospital admission" OR "hospital admission" OR "admitted to hospital" OR "admitted to hospitals").ti,ab. | 253066 |
| 3  screening | (screening OR screened).ti,ab. | 788263 |
| 4 | 1 AND 2 AND 3 | 914 |
| 5 | 4 AND ("systematic review".pt. OR ("systematic review" OR "systematic reviews" OR "scoping review" OR "scoping reviews" OR "umbrella review" OR "umbrella reviews").ti.) | 76 |

Research Questions Included:

How complete and accurate are documentation of race and ethnicity in observational health databases, including EHRs?

What SDOH data elements are included in observational health databases, including EHRs?

| **Set #** | **Search Strategy** | **Results** |
| --- | --- | --- |
| 1 | ("social determinant" OR "social determinants" OR race OR racial OR ethnicity OR ethnicities OR "minority group" OR "minority groups" OR sociodemographic OR socioeconomic OR "social class" OR "social factor" OR "social factors" OR "education level" OR "education levels" OR urban OR rural OR pollution OR immigration OR "undocumented immigrant" OR "undocumented immigrants" OR "drug use" OR "drug abuse" OR "abusing drugs" OR "alcohol use" OR alcoholic OR alcoholics OR incarceration OR incarcerated OR prisoner OR prisoners OR gender OR genders OR sexuality OR sexualities OR "sexual orientation" OR "sexual orientations" OR "military personnel" OR veteran OR veterans OR "economic stability" OR income OR housing OR workplace OR "work place" Or transportation OR "area index deprivation" OR geographic OR environment OR ability OR abilities OR walkability OR walkable OR park OR parks OR education OR language OR languages OR diet OR diets OR food OR foods OR family OR families OR community OR communities OR "social context" OR "social integration" OR women OR "mental health" OR insurance OR insured OR "provider availability" OR "social vulnerability index" OR "deprivation index").ti,ab. | 6646725 |
| 2  health databases | ((medical OR electronic OR patient OR surveillance) ADJ2 (based OR record OR records OR card OR cards OR claim OR claims OR waiver OR waivers OR data OR database OR databases)).ti. OR (ehr OR ehrs OR emr OR emrs OR "health database" OR "health databases").ti. | 35759 |
| 3  completeness/accuracy terms | (complete OR completely OR completeness OR accuracy OR accurate OR inaccurate OR inaccuracy OR inaccuracies).ti,ab. | 2028605 |
| 4 | 1 AND 2 AND 3 | 1594 |
| 5 | 4 AND ("systematic review".pt. OR ("systematic review" OR "systematic reviews" OR "scoping review" OR "scoping reviews" OR "umbrella review" OR "umbrella reviews").ti.) | 41 |
