# Supplementary material for: Evidence gaps among systematic reviews examining the relationship of race, ethnicity, and social determinants of health with adult inpatient quality measures
Source: Antimicrob Steward Healthc Epidemiol. 2024 Sep 23;4(1):e139. doi: 10.1017/ash.2024.397 (PMC11427999; doi:10.1017/ash.2024.397)

**Supplement 2. Assessing quality of our narrative review with the Scale for the Assessment of Narrative Review Articles (SANRA)**


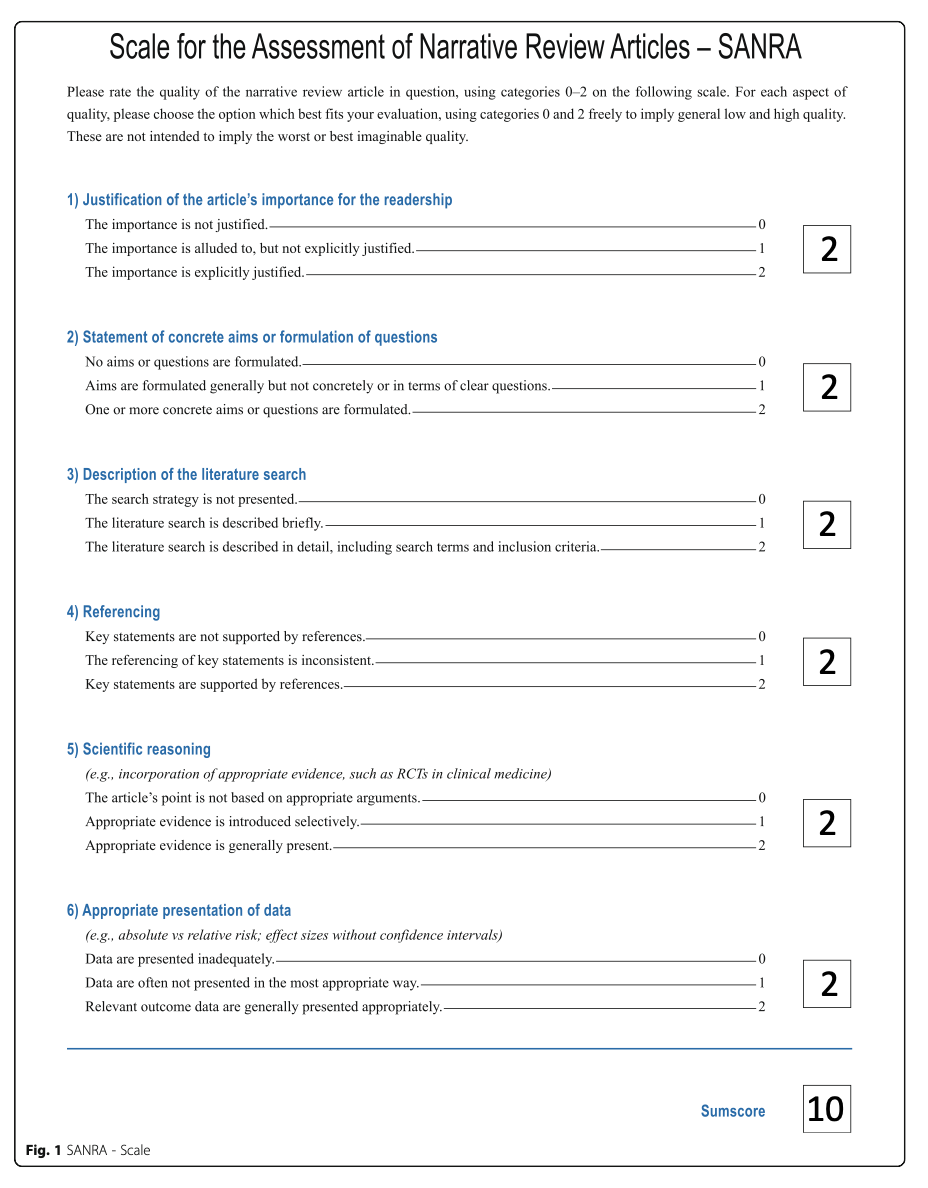

Supplement: Advani et al. supplementary material 2 — Advani et al. supplementary material [file S2732494X24003978sup002.docx]
